# The Down Low Among Latino Men Who Have Sex With Men: A Review and Theoretical Analysis

**DOI:** 10.7759/cureus.108108

**Published:** 2026-05-01

**Authors:** Keith R Head

**Affiliations:** 1 Social Work, Independent Researcher, Lubbock, USA; 2 School of Nursing and Health Sciences, Capella University, Minneapolis, USA

**Keywords:** bisexuality, concealment, down low, familismo, hiv, latino, machismo, sexual identity, sexual nondisclosure, sexual risk

## Abstract

Being on the "down low" (DL) describes men who identify as straight and secretly engage in same-sex encounters while maintaining heterosexual public identities. While the DL phenomenon has been discussed primarily in the context of African American men, Latino men in the United States experience similar patterns of sexual concealment shaped by cultural forces not experienced similarly by other groups. Relatively little is still known about how machismo, familismo, and religious values affect the sexual decisions and health outcomes of Latino men who have sex with men (MSM), and most existing research aggregates Latino MSM with other racial and ethnic minorities without examining their experiences separately. This article applies three theoretical frameworks to the DL among Latino men. The concealment-specific model is used to explore how the act of hiding same-sex behavior produces anxiety, depression, and ongoing self-monitoring, particularly within cultural settings where discovery threatens masculine standing and family belonging. Sexual configuration theory is used to examine why Western categories of gay and bisexual do not fit the way many Latino men understand their own sexuality, especially in cultural contexts where sexual role rather than partner gender defines sexual identity. Syndemic theory is used to examine how conditions such as homophobia, HIV stigma, substance use, poverty, and immigration status interact to worsen health outcomes for this population. The findings of this analysis suggest that DL behavior among Latino men is shaped by the interaction of cultural, psychological, and structural conditions and cannot be understood through any single framework alone. Applied in combination, these theories explain DL behavior among Latino men more fully than established Western approaches. This article is intended to support future empirical research and the development of culturally informed interventions for Latino MSM.

## Introduction and background

Introduction

The term "on the down low" (colloquially DL) refers to the phenomenon of men who secretly engage in same-sex sexual behaviors while presenting as heterosexual or not openly identifying as gay or bisexual. Though the expression first arose within African American vernacular during the mid-1990s as a term for any behavior done with discretion, it gained widespread mainstream attention following J.L. King's 2004 appearance on The Oprah Winfrey Show. During the course of one hour, King testified to his own 25-year experience of existing on the DL as a secretly bisexual Black man, describing how desire "overrode everything" and "created this whole secret life" that ultimately destroyed his family [1]. The episode, and the media frenzy that followed, fixed the DL phenomenon squarely within African American communities in public consciousness. What was largely overlooked, and what remains understudied, is the extent to which Latino men in the United States navigate a parallel dynamic shaped by cultural forces that operate quite differently from those documented in the Black DL literature.

In Latino communities, men who have sex with men (MSM) often uphold outward heteronormative roles while maintaining relationships with women and secretly engaging in sexual encounters with men, a pattern shaped by cultural expectations around masculinity, family belonging, and the discretion surrounding non-heteronormative behavior [2]. The DL identity is not openly gay or bisexual; rather, it is characterized by nondisclosure and discretion [3]. Many MSM in racial/ethnic minority groups, including Latinos, do not disclose their same-sex behavior or orientation, often to avoid social isolation, discrimination, or abuse [1]. This nondisclosure has serious public health and social implications. The US Centers for Disease Control and Prevention (CDC) found that MSM who hide their sexuality are more likely to engage in high-risk behaviors and can become an important bridge for HIV transmission to female partners [4]. The existing literature has tended to aggregate Latino MSM with Black and other racially diverse MSM without separating results by ethnicity, a practice that obscures the distinct cultural and behavioral dynamics at work [5,6]. Muñoz-Laboy argued that the MSM category itself is insufficient to capture the multidimensional aspects of Latino male bisexuality and that many men do not identify with the label, contributing to their alienation from scholarly inquiry and prevention strategies alike [3].

These gaps in the literature are not merely academic. Only 17% of peer-led HIV prevention interventions have been deeply culturally adapted for Latino MSM populations, and longitudinal research examining how DL behavior changes over the life course is virtually nonexistent [7]. Most existing interventions have been developed for openly gay-identified men and then superficially adapted for use with non-disclosing populations whose relationship to sexual identity is fundamentally different. Research on Latino cultural factors has often reinforced a heteronormative perspective by focusing primarily on gender and nationality while neglecting sexuality and sexual orientation entirely [8]. The result is a body of research that treats Latino men on the DL as a footnote to a phenomenon understood primarily through the experiences of other communities.

This review addresses that gap by synthesizing a literature that has remained fragmented, largely aggregated with other minority MSM populations, and weighted toward the Black DL experience, examining how cultural factors, including machismo, familismo, religious orthodoxy, and immigration status, create conditions where nondisclosure becomes a response to environmental pressures rather than a pathological behavior requiring correction. The analysis then applies complementary theoretical frameworks to explain the mechanisms sustaining concealment, the cultural production of sexual identity categories, and the interaction of forces that compound health vulnerabilities among Latino men on the DL. Rather than pathologizing men who live on the DL, this review takes the position, consistent with other scholars, that the real targets of intervention should be the cultural and structural conditions that make concealment feel necessary in the first place.

Background

The practice of men engaging in same-sex sexual behavior while identifying publicly as heterosexual is not new. In 1961, Albert Reiss was among the first to note that some men in the United States who engage in same-sex behavior do not consider themselves homosexual [9]. Parallel patterns were documented among Mexican and other Latino men well before the DL terminology emerged, though this history has received far less attention [10,11]. What is comparatively recent is the emergence of a specific cultural identity and vernacular surrounding this behavior, and the public anxiety it has generated. The term "down low" first arose during the 1990s among African Americans as an adjective meaning covert or secretive. The acronym DL meant "keep secret or hidden" for teenagers in the United States, and for African Americans more broadly, it referred to something kept very quiet, done on the sly [5]. The term appeared in rhythm and blues songs as an indicator of male infidelity, notably R. Kelly's 1996 track "Down Low (Nobody Has to Know)," and circulated in other forms of popular culture, primarily in Black male rap, as a marker of a man keeping his business to himself. The sudden ominous image of a "straight" Black man engaging in secret sexual activity with male partners was a relatively new and narrower characterization of the term. Alongside this construction, men began to adopt "DL" as an identity label, used for instance in self-descriptions in personal ads on the web, where it indicated both a desire for privacy and a marker of masculinity and sexual prowess [1].

The DL entered mainstream public consciousness through a series of media events in the early 2000s. On August 3, 2003, the New York Times Magazine published an extensive report describing what it called "an organized, underground subculture largely made up of Black men who otherwise live straight lives" [12]. Even nearly a decade earlier, author E. Lynn Harris had written bestselling novels describing the phenomenon of Black male bisexuality. However, the moment that crystallized public attention was J.L. King's appearance on The Oprah Winfrey Show on April 16, 2004, in an episode titled "A Secret World of Sex: Living on the 'Down Low.'" King, a self-declared HIV prevention activist, bore witness to his own 25-year experience of living on the DL as a secretly bisexual Black man, and heavily publicized his memoir, marketed as the first thorough investigation of life on the DL. The episode generated substantial public alarm. The anxiety and anger resulting from the "discovery" of this supposedly new phenomenon inspired a proliferation of self-help books, blogs, and media segments recommending ways to identify men on the DL and advising women who suspected their partners of secretly engaging in sex with men [1].

From the outset, the popular construction of the DL was fraught with complexities and contradictions. A clear and consistent definition was noticeably lacking. The phenomenon was portrayed as something new, despite the fact that the existence of male bisexuality has been well-documented across cultures since ancient times [1,13]. The cyclical erasure, stigmatization, re-erasure, and re-stigmatization of bisexuality, particularly among men, was hardly new [14,15]. What was different this time was the framing. Previous media depictions of bisexuality had portrayed it as a glamorous trend among pop stars and supermodels. The DL, by contrast, was constructed as a shameful secret that put innocent people, women in particular, at risk for disease and death [1]. The phenomenon was elevated by some to the status of a driving force behind the HIV epidemic in the Black community, despite the absence of direct empirical evidence supporting this claim [16]. Through what Loseke identified as a "villain and victim frame," media coverage constructed men on the DL as unrepentant villains and heterosexual women as unsuspecting victims of horrifying consequences, a narrative structure that proved effective at sustaining public attention [17,18].

Perhaps most consequentially, the discourse surrounding the DL characterized secretive bisexuality as being all but exclusive to Black men. This represented an abrupt about-face in terms of racial focus, as classical studies of bisexuality in the United States had focused heavily on White men and women, and early images of the "bisexual bridge" from bisexual male-to-female partners promulgated the stereotype of the White, married man as a vector of disease transmission [1]. The sudden racialization of the phenomenon resulted in renewed demonization not only of bisexuality but of Black male sexuality specifically [19]. In the rush to frame the DL as a Black problem, the rage caused by the alleged bisexuality of men of color contrasted sharply with the historical silence on White men's bisexuality. Although most press descriptions of the DL included young Latino men, Latino communities, where sexual nondisclosure is shaped by distinct cultural logics around masculinity, family, and sexual role, have largely eluded this discussion [5]. This omission is notable given that men engaging in same-sex behavior without adopting gay or bisexual identities had been documented among Mexican men decades earlier [10].

An important distinction often lost in both media coverage and scholarly discourse is that the DL is not simply a closeted form of gay or bisexual identity. Men on the DL testify to having heterosexual orientations, and terms such as homosexual or bisexual do not aptly apply because these men are not concealing an identity they privately hold but rather operating under a framework in which their same-sex behavior does not define their sexual identity in the first place [17,20]. Research with DL men has found that they react negatively to the gay label, not because of the object of their sexual desire but because of what the label culturally signifies, associating it with weakness, emotionality, and femininity [21]. For Latino men in particular, "gay" carries race, class, and gender implications distant from their social, political, and cultural locations, and rejecting gay identity does not mean relinquishing membership in their ethnic community or their political life within it [5]. Treating these men as simply gay but not yet self-aware contributes to bisexual erasure and prevents researchers and practitioners from engaging the population on its own terms [22].

Importantly, DL behavior is not confined to any single racial or ethnic group. Data from the Urban Men's Health Study found support for DL behavior among both Black and Latino men, with non-White men who identify as heterosexual or "something else" comprising an estimated 8.2% of the MSM sample, compared to 6.3% for White men [17]. Rates of behavioral and self-identified bisexuality in large MSM samples have consistently been found to be highest among ethnic minority men, and Latino MSM in particular are less likely to identify as "gay," less likely to join gay-related organizations, and less likely to read gay-related media than their White counterparts [1,16,23]. Among Latino men specifically, a multi-state survey of 1,482 Latino MSM found that 62% did not identify as gay [24]. The phenomenon cuts across racial and ethnic lines but takes particular forms in particular communities, shaped by distinct cultural forces that the following sections examine in detail.

## Review

Methodology

A narrative review of existing literature was conducted to examine how cultural factors, such as machismo, familismo, religious orthodoxy, and immigration status, sustain sexual nondisclosure among Latino MSM in the United States. Three complementary theoretical frameworks were then applied to the phenomenon: the concealment-specific model, sexual configuration theory (SCT), and syndemic theory.

To identify relevant sources, a literature search was conducted using academic databases, including PsycINFO, PubMed, ERIC, CINAHL, SocINDEX, and Google Scholar. Search terms included combinations of terms such as "Hispanic", "Latino", or "Latinx" with "men who have sex with men", "MSM", "down low", "bisexual behavior", "nondisclosure", "concealment", "machismo", "familismo", "stigma", "religion", "immigration", "HIV", "sexual identity", and "syndemic". The search was limited to sources in English and Spanish published between 2000 and 2026 and included earlier foundational texts when they clarified historical context or conceptual evolution. Inclusion criteria required a focus on US-based Hispanic or Latino populations or on the origins and cultural construction of DL behavior across racial and ethnic groups, with attention to at least one core domain of the analysis. Peer-reviewed empirical studies, systematic reviews, and clinical guidelines were prioritized, supplemented by scholarly books, dissertations, and authoritative reports when they directly informed the theoretical analysis. Sources without cultural specificity to the populations under review, materials that did not differentiate findings by race or ethnicity, and non-scholarly media were excluded from the empirical evidence base, though selected media sources were used to illustrate contemporary public discourse. Terminology surrounding these behaviors varies across academic, public health, and popular contexts. "Men who have sex with men" functions as a behavioral and epidemiological classification, while "down low" or "DL" carries cultural and identity-related connotations and remains the most widely recognized term in mainstream discourse. The search spanned both clinical terminology, such as MSM, and colloquial terminology, such as DL, to ensure comprehensive retrieval.

No new data were collected, and no human subjects were directly involved, as this study relies exclusively on secondary data from published sources. As such, IRB review was neither required nor sought. This narrative review was conducted in alignment with the Scale for the Assessment of Narrative Review Articles (SANRA) [25].

Cultural and structural context

Cultural Norms, Masculinity, and "Machismo"

Machismo enforces a version of masculinity that makes same-sex desire incompatible with social standing. The expectations it produces, including hypermasculinity, emotional restraint, dominance, and sexual prowess, create a framework in which homosexual behavior, particularly when it implies a feminized role, is stigmatized as failed manhood [26,27]. In Latino culture, masculinity is associated with being the penetrative partner in sex, serving as a financial provider, and exhibiting emotional stoicism [28]. Failure to fulfill these gender scripts generates doubts about sexual preference, and men are defined not only in contrast to women but in opposition to subordinated masculinities.

The ethnographic literature on sexuality in Latin America concurs that sexual identity in many contexts is organized not around the gender of one's partner but around the sexual role one assumes. Among Mexican and Dominican men, the insertive partner (the "activo") may consider himself heterosexual, while the receptive partner (the "pasivo") is labeled homosexual [1]. Carrier [10] and Taylor [29] documented that Mexican men categorized same-sex encounters on this active/passive distinction, allowing men to have sex with other men without adopting a gay or bisexual identity so long as they performed the penetrative role. Nesvig argued that this creates a "complicated terrain" for Latino men's sexuality, in which men reject gay or bisexual labels while engaging in same-sex activity under a cultural model that preserves their masculine status [11]. Masculine Latino MSM can navigate the sexual/gender system, avoid stigma, and pass as heterosexual. It is overly simplistic to infer that their failure to adopt a "gay" identity implies internalized homophobia; rather, familiar Anglo-American categories of sexual orientation are culturally specific, and their imposition on Latinos is problematic.

These dynamics persist among Latino MSM in the United States. Halliday proposed that ethnic minority MSM are influenced by a trifecta of cultural pressures not experienced similarly by White MSM, including expectations to achieve different standards of masculinity, compounded stigmas associated with minority status, and more frequent involvement in interdependent family models [20]. Minority males may feel they have to exaggerate masculine attributes to compensate for a lack of privilege that comes with not being White, and overt displays of physical masculinity may be perceived as a route through which masculine status can be earned [20]. Ocampo found that Latino men who engaged in same-sex behavior described actively "doing" masculinity, deepening their voices, talking about dating women, and exaggerating heteronormative behavior to avoid negative judgment [30]. In a multi-state survey of 1,482 Latino MSM, 62% did not identify as gay, with many identifying as heterosexual or "nothing" despite same-sex activity [24]. This gap has been attributed partly to cultural expectations in which openly identifying as gay threatens masculine social standing while maintaining a heterosexual identity permits men to function in their social worlds without the stigma attached to homosexuality [31]. Adopting a queer identity may reduce an ethnic MSM's perceived sense of masculinity, which is intrinsic to maintaining social standing where privilege is already lacking [20]. For many Latino men, coming out implies making hard choices that prioritize the individual over the communal self, and it should not be surprising that some decide to keep their sexuality on the DL.

Family Expectations and "Familismo"

Familismo ties the benefits of family belonging to heteronormative conformity. The interdependence, loyalty, and solidarity that define familismo come with the expectation that men will marry women, produce children, and protect familial honor, and the prospect of a son coming out threatens all of these simultaneously. Many Latino gay and bisexual men fear that disclosure will be seen as a betrayal of family loyalty [26,32].

The strong ties within Latino families can become a major source of conflict for gay and bisexual men. Familismo values, as strong in Latino gay and bisexual men as in any other members of the culture, prevent them from denouncing the family's homophobia and demanding acceptance. Instead, for the sake of psychological connectedness and identification with the family, homophobia tends to be internalized in a self-punitive way [33]. The protections of familismo are contingent upon restrictions; to receive the benefits of the network, individual members must conform to its social norms and expectations.

Muñoz-Laboy, in an ethnographic study of bisexually active Latino men in New York City, found that familismo operates at the societal level to control public imagery of sexuality and gender conformity [3]. Two themes were strongly expressed in the narratives. First, participants described wanting to procreate and have a family while protecting familial honor by keeping their bisexuality outside the realm of their families. Some men rushed into early marriages or pursued pregnancies to demonstrate dedication to family and foreclose suspicion about their sexuality. Second, familismo promotes a compartmentalization between the familial life and the sexual-erotic-romantic life that is not necessarily imposed on the heterosexual counterparts of bisexual Latino men. The strategy of compartmentalization precipitates emotional disconnectedness among those who desire to share with their families the emotional aspects of their bisexuality. One research participant in that study expressed, "my idea is not to have them [relationships with men]…because of the moral, because of my family, my son, I just want to have a normal life" [3].

The family's influence extends into material reality. Latino men embedded in interdependent family models derive emotional, social, and financial well-being from family connections, and disclosure threatens all of these [20]. As one participant in Martinez et al. explained, he stayed silent because "I have my family … they [would] make fun of you… There is no respect" in the community for openly gay or bisexual men [31]. Diaz described this as "sexual silence," a broader cultural tendency to avoid discussions of sexuality altogether, particularly anything non-heteronormative [33]. Some families operate under an implicit arrangement in which, so long as a son or husband does not openly disclose, the family does not acknowledge what may be apparent. Secrecy within Latino families functions as a strategy to negotiate acceptance; as long as men do not verbalize references to same-sex relationships or attraction in familial settings, there is no conflict, even if they have previously come out. Muñoz-Laboy further found that familismo imparts a deep notion of shame and guilt on non-heterosexual sexual expressions and that men deeply embedded in family networks may avoid behaviors their community stigmatizes, viewing DL behavior as responsible conduct that protects the family's well-being [3]. While familismo can serve as a protective factor when families are accepting, in the context of DL culture, it more often operates as a conservative force enforcing heteronormativity.

Religion, Morality, and Homophobia

Religious institutions in Latino communities function as a second enforcement mechanism alongside the family, reinforcing heteronormative expectations through moral authority. A large proportion of Latinos are raised in Catholic or evangelical traditions that treat homosexual acts as sinful, and community churches anchor heterosexual marriage and child-rearing as spiritual obligations rather than mere social preferences [34,35]. The Catholic Church distinguishes inclination from behavior, viewing same-sex attraction as disordered but not sinful, while treating acting on those desires as sin. This nuance is largely lost in practice, and many Latino Catholics absorb the broader message that homosexuality in any form is morally wrong. Marianismo, rooted in the ideal of the Virgin Mary, idealizes women as pure and family-focused, indirectly reinforcing the sanctity of traditional heterosexual family structure for men as well [36].

For Latino men with same-sex attraction, religious upbringing can generate significant conflict between spirituality and sexuality. García et al. found that Latino gay men spoke of internalizing messages that homosexuality is "abnormal" or "an abomination," leading them to reject or conceal their orientation to remain in good standing with their faith and family [35]. Some attempted to suppress their desires through prayer or church involvement, while others attended services as a dutiful family member and kept their private sexual lives separate [35,37]. In some Latino immigrant communities, homosexuality is perceived as taboo and as something that "does not happen to us" but rather as a behavior belonging to Americans or other outside groups, a denial that can lead to harsh treatment of those in the community who come out [38].

Not all Latino families are uniformly unaccepting, and attitudes are slowly changing, particularly among younger generations and in more secular households. Nonetheless, exposure to non-affirming religious environments has been found to correlate with higher internalized homophobia among LGBTQ individuals. Barnes et al. reported that, in a national sample, Latino LGBTQ people had some of the highest levels of internalized homophobic attitudes, linked to frequent exposure to religious anti-gay messages [39]. This internal conflict, the belief that one's desires are sinful or wrong, makes it difficult for men to embrace an openly gay or bisexual identity, and silence and secrecy become the means through which they manage the contradiction.

Stigmatization and Moral Blame of HIV/AIDS

Latino MSM are disproportionately affected by HIV, accounting for 39% of estimated new infections among all MSM in 2022, despite Hispanics comprising 18% of the US population [40]. Stigma related to HIV and STIs represents an additional factor that impacts DL behavior among Latino MSM. The fear of being associated with HIV deters testing and engagement with prevention services, as men worry that seeking testing will be interpreted as confirmation of stigmatized sexual behaviors [41]. Latino men have been found to avoid testing facilities out of concern that being seen at such locations would lead others to presume they are gay or promiscuous, exposing hidden same-sex activity to family and community [27]. This anxiety is compounded by cultural taboos in which discussions of sex are considered shameful, making it difficult for men to seek sexual health information without arousing suspicion. Latino MSM experience both HIV-related stigma and sexual minority stigma simultaneously, creating a dual barrier to accessing care that is less prominent among White MSM populations [42].

Latino MSM have been found to report higher levels of HIV stigma compared to other racial groups, and this elevated stigma correlates with lower testing rates, reduced condom use, and decreased familiarity with prevention methods such as pre-exposure prophylaxis [42]. The stigma extends to disclosure practices. Carballo-Diéguez et al. found that HIV-positive Latino MSM were significantly less likely to disclose their serostatus to sexual partners, with 41% acknowledging that they had misrepresented their status to prospective partners met online [43]. Because the association between HIV and homosexuality in the public consciousness means that any HIV-related concern threatens to expose a man's sexual identity, some men continue risky sexual practices rather than seek care that might require them to acknowledge same-sex behavior to providers or explain their need for testing to family [44]. The code of silence that serves as a strategy for mitigating stigma in familial networks is reproduced in medical contexts, where men avoid disclosing their sexual orientation or sexual behavior to avoid possible discrimination.

Immigration and Acculturation Factors

Immigration introduces additional complications for sexual identity among Latino men. Latino immigrant MSM are less likely to identify as gay or bisexual compared to US-born Latino MSM, a difference attributed to upbringing in societies with less tolerance for LGBTQ individuals and the persistence of those norms after migration [45]. Men who grew up in rural Mexico or El Salvador may carry those attitudes with them when they relocate, and even when the US environment offers greater openness, familiar patterns of concealment tend to hold.

Immigrants often settle in tight-knit Latino communities where social life revolves around family, church, and community gatherings, and these communities are often densely networked, with information circulating rapidly among members. Martinez et al. found that Latino bisexual men in the Midwestern US perceived the local Latino community as "very closed" regarding sexual diversity and that fear of gossip kept them from confiding in anyone [31]. Language barriers and limited access to LGBTQ resources compound this. In emerging Latino communities in new destinations such as the Midwest or rural South, there is a lack of culturally congruent public health and community resources for Latino bisexual and MSM individuals [31]. Services with Spanish-speaking staff or culturally competent outreach are scarce, leaving immigrant men without counseling, HIV testing, or support groups where they might discuss sexuality.

Migration also creates situational conditions for DL behavior. Latino men who migrate without their families, particularly solo male labor migrants, may engage in same-sex activity during periods of separation from wives or partners. González-López documented reports of Mexican migrant workers having sexual encounters with other men while working away from their families, yet continuing to identify as heterosexual [46]. The migration context removes men from their home community's watchful eyes, while their identity remains rooted in traditional expectations.

Intersectionality

Latino men on the DL may experience unique stressors associated with their overlapping identities [38]. They confront homonegativity within their own ethnic community while experiencing racism within mainstream society, which is predominantly White. When sexual and ethnic minority status are coupled, Latino gay men experience both racism and homonegativity from mainstream society and hegemonic masculinity within their own community. Navigating these dual identities may be difficult for some men as they attempt to understand what it means to belong to both groups that have been historically marginalized.

Within Latino communities, slurs such as "maricón," "joto," and "pato" function to police gender conformity and disparage those perceived as homosexual, and the threat of being labeled with these terms deters disclosure. Community gossip amplifies the stakes. One participant in Martinez et al. described saying he "never talks with anybody" about his situation and instead retreats to his room to talk with himself about whether what he is doing is wrong [31].

In mainstream LGBTQ spaces, Latino men encounter a different set of barriers. If mainstream queer culture is built around and more receptive to the affluent White male, it is more difficult for ethnic minorities to identify within that structure because their cultural values are not fully represented [20]. Ethnic minority MSM find it difficult to relate to mainstream gay culture because they view it as primarily White and feminine [1]. Latino men often do not see themselves reflected in gay media or find that their cultural needs, including bilingual support, are unmet.

Martinez et al. also documented this among bisexually active Latino men in Indianapolis, where all 25 participants reported a lack of a visible bisexual or gay Latino community and felt they did not belong to any group. One man stated, "People being bisexual, we are really depressed because we feel that they don't belong to any group" [31]. Latino LGBTQ individuals report higher overall rates of discrimination than their White counterparts, with 46% reporting discrimination compared to 31% of White LGBTQ adults [47]. When a man perceives that disclosure will alienate him from his family without acceptance elsewhere, remaining on the DL becomes a way to manage an environment in which no available identity offers full belonging.

Theoretical analysis

The cultural and structural conditions already documented describe what Latino men on the DL navigate, but do not explain the mechanisms through which those conditions produce their effects. Three theoretical frameworks address this gap, each engaging these phenomena from a different angle.

The Concealment-Specific Model

Pachankis's concealment-specific model deals with what happens psychologically when men sustain the kind of nondisclosure described in the preceding section. The model argues that individuals who conceal a stigmatized identity engage in ongoing cognitive preoccupation, hypervigilance, and behavioral modification that produce psychological harm whether or not the concealed identity is ever discovered [48]. What sets concealment apart from other forms of minority stress is that the person carries the full weight of managing the boundary between public knowledge and private practice, and this management is not a one-time decision but a sustained effort.

The machismo-driven self-monitoring that Ocampo described, in which men deepen their voices, make up stories about women, and exaggerate heteronormative behavior, maps onto what Pachankis calls anticipatory processing [30]. These men are doing the mental work of predicting which situations could expose them and rehearsing how to respond. The men that Muñoz-Laboy documented, who rushed into marriages or pursued pregnancies to head off suspicion, were engaged in what the model calls preventive behavior [3]. The problem is that each preventive action makes future disclosure harder by adding relationships and obligations that would have to be undone.

The participant in Martinez et al., who retreated to his room to talk to himself about whether what he was doing was wrong, illustrates another dimension of the model [31]. In Pachankis's terms, concealment forces the individual into a self-evaluative loop where, because he cannot get feedback from anyone else, he ends up acting as both prosecutor and defendant with no resolution [48]. The sexual silence that Diaz [33] identified within Latino families makes this worse. When sexuality is never discussed, the man hiding his behavior has no way of testing whether disclosure would actually bring the consequences he fears [33]. The silence feeds the concealment, and the concealment feeds the silence.

This dynamic extends into medical settings. Men who avoided HIV testing because being seen at a clinic might expose their sexual behavior were not just responding to HIV stigma on its own. Any health-seeking behavior that could signal same-sex activity threatened the entire identity they had built. The model would predict precisely this pattern, that men managing concealment will avoid situations where they might have to reveal something about their sexual behavior, even when avoidance puts their health at serious risk, because the fear of exposure sits closer to the surface than the fear of disease.

Sexual Configuration Theory (SCT)

SCT, developed by van Anders, offers a way of thinking about the activo/pasivo framework that does not require treating it as a deviation from how sexual identity is supposed to work [49]. Where the ethnographic literature shows that many Latino men organize sexual identity around penetrative role rather than the gender of their partner, SCT explains why this is not a failure to adopt the right categories. The theory breaks sexual identity apart into independent dimensions, including partner gender, sexual behavior, erotic identity, and gender expression, and treats each one as its own axis rather than folding them all into a single orientation label [49].

Under this framework, the rejection of gay identity documented among Latino MSM in the results stops being a contradiction that needs explaining. These men are working within a system where the behavioral dimension (penetrative role) and the identity dimension (heterosexual masculinity) run along different lines than the Anglo-American model assumes. Carrier [10] and Taylor [29] showed that the activo retains heterosexual status because, within this system, it is what a man does in sex and not who he does it with that determines his sexual identity. SCT gives theoretical grounding to what the ethnographic record has been saying for decades, that this is a coherent way of organizing sexuality and not simply a refusal to come to terms with being gay.

The theory also speaks to why Latino MSM reported feeling excluded from mainstream LGBTQ spaces. If that culture organizes identity around partner gender preference, then men whose sexuality runs along role, penetrative behavior, and masculine presentation are not going to see themselves in it. The men in Martinez et al. who said that they belonged to no group were not just socially isolated. They fell between identity systems [31]. Neither the heteronormative Latino community nor the mainstream gay community had a category that fit them. SCT reframes this from a problem of the individual to a problem of the available categories. When Nesvig wrote about the "complicated terrain" of Latino men's sexuality, the complication was in the analytic tools being used, not in the men's own experience [11].

Syndemic Theory

Syndemic theory, first articulated by Singer [50] and applied to MSM health by Stall et al. [51], explains why the conditions described in the results produce outcomes worse than any one of them would on its own. A syndemic is not just several health problems happening at the same time. It is the interaction between them, where each one makes the others more severe, within a population that structural inequality has already made vulnerable. The conditions have to be understood as reinforcing each other rather than simply piling up, and the social and structural forces behind the vulnerability deserve as much attention as the health outcomes themselves [50,51].

The material in the results fits this pattern clearly. Machismo enforces emotional stoicism, which discourages help-seeking, which leaves depression and anxiety untreated, which increases the likelihood that men will turn to substances to cope, which lowers inhibition around sexual risk, which increases HIV exposure, which generates more stigma, which reinforces concealment, and which deepens the conditions that started the cycle. No single link in that chain is enough to account for the health burden that Latino MSM carry. It is the way the links feed back into each other that produces the disproportion.

Immigration status, language barriers, poverty, and the absence of culturally competent services are not just the context for these dynamics. They are part of the syndemic. A man who avoids HIV testing because it might reveal that his sexual behavior is in a very different situation if he is also undocumented, uninsured, speaks only Spanish, and lives somewhere without bilingual health services. The theory predicts that addressing any one of these barriers in isolation will have a limited impact because the others compensate. The emerging Midwestern Latino communities described earlier illustrate this point, where even men who wanted sexual health information had no way of getting it without risking exposure. Table 1 summarizes each framework by level of analysis, the DL dynamic explained, and the proposed mechanism.

**Table 1:** Theoretical frameworks applied to DL dynamics among Latino MSM DL: down low; MSM: men who have sex with men

Framework	Level of analysis	DL dynamic explained	Mechanism
Concealment-specific model (Pachankis [48], 2007)	Individual/psychological	Machismo-driven performance of heterosexuality	Anticipatory processing; men rehearse responses and monitor behavior to avoid exposure
		Rushing into marriage or fatherhood to prevent suspicion	Preventive behavior that raises the cost of future disclosure
		Isolated self-interrogation within sexually silent families	Self-evaluative looping without external feedback to interrupt it
		Avoidance of HIV testing and clinical settings	Health-seeking behavior perceived as a threat to concealed identity
Sexual configuration theory (van Anders [49], 2015)	Conceptual / identity	Activo/pasivo organization of sexual identity by role rather than partner gender	Behavioral and identity dimensions operate on independent axes
		Latino MSM not identifying as gay despite same-sex behavior	Western orientation labels collapse dimensions that function separately in Latino sexual systems
		Exclusion from mainstream LGBTQ spaces and the absence of community belonging	Identity organized around role and masculinity does not map onto partner-gender-based gay culture
Syndemic theory (Singer [50], 1996; Stall et al. [51], 2003)	Structural/epidemiological	Feedback chain from machismo through substance use, sexual risk, HIV exposure, and back to concealment	Each condition worsens the next; outcomes exceed what any single factor would produce
		Immigration status, language barriers, and poverty compounding sexual health risk	Structural barriers are embedded in the syndemic, not the background to it
		Dual stigma foreclosing access to both the ethnic community and the gay community support	Each potential source of support requires disclosure that the other source penalizes

An Integrated Approach

The three theories converge on a shared set of phenomena, but each explains different aspects of them. The concealment-specific model explains the internal costs of hiding. SCT explains why the identity categories available to these men do not fit their experience. Syndemic theory explains why the environment they live in makes it impossible to resolve either the psychological burden or the categorical problem in isolation.

**Figure 1:**
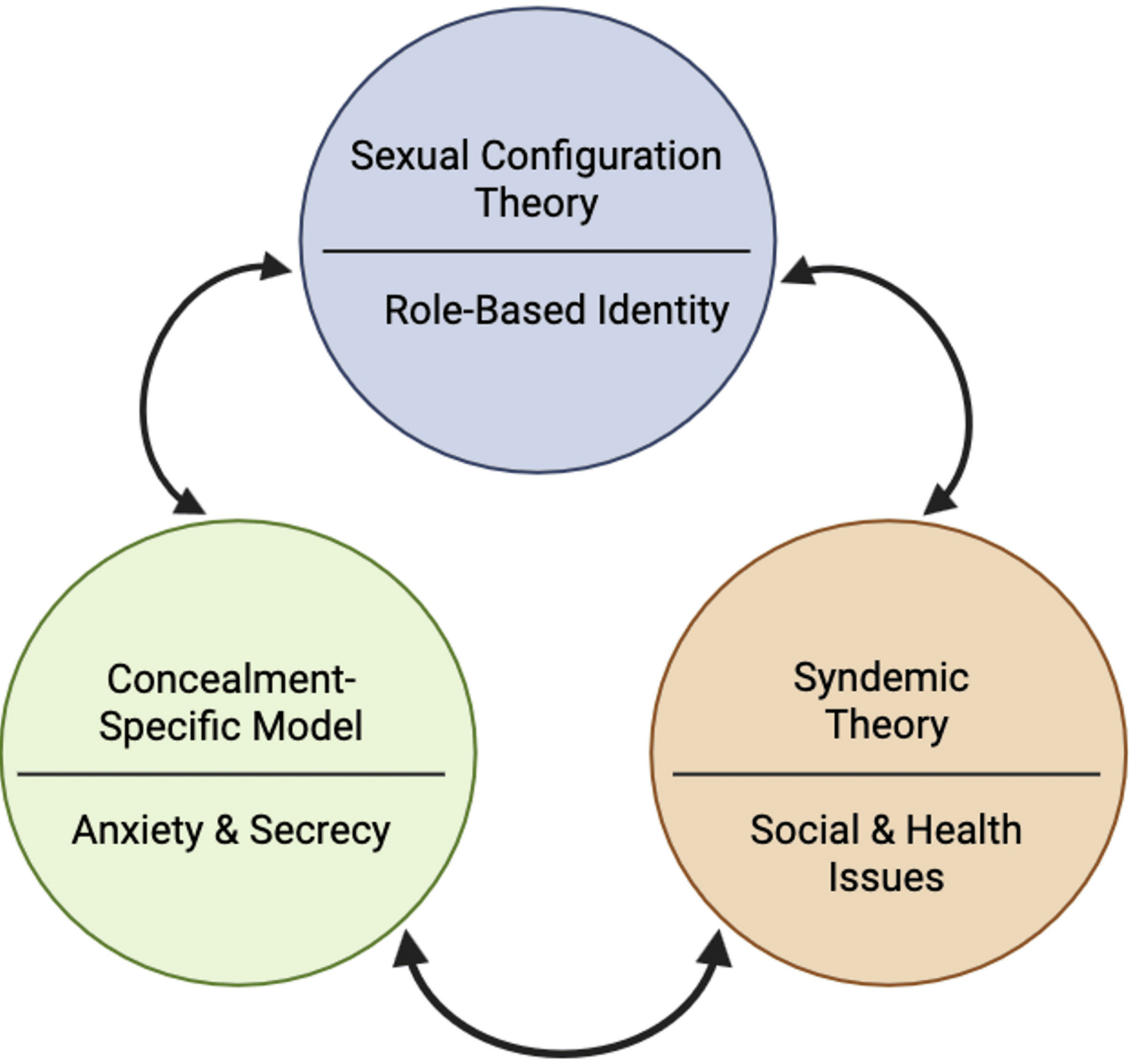
Integrated frameworks for understanding DL Latino MSM Created by the author (Head K, 2026) in BioRender.com (BioRender, Toronto, ON) DL: down low; MSM: men who have sex with men

The bind that Halliday described shows the convergence in concrete terms [21]. A White gay man dealing with homophobia can seek support in a gay community built around his cultural norms [20]. A straight Latino man dealing with racism can fall back on family and ethnic community. A Latino man on the DL cannot access either without exposing what he is hiding. The stress that comes from hiding is compounded by the fact that the identity categories SCT identifies as inadequate are also the only ones available to these men, while the structural conditions Singer [50] describes keep pushing them back into concealment rather than opening alternative paths. Addressing any one level without the others is unlikely to change outcomes. The psychological costs of concealment, the inadequacy of existing identity categories, and the structural conditions that make concealment feel necessary all have to be engaged together (Figure 1).

Discussion

This review examined how machismo, familismo, religious values, and immigration shape sexual nondisclosure among Latino MSM and applied three complementary frameworks to explain the mechanisms involved. The discussion that follows considers how Latino nondisclosure differs from the Black DL narrative, the role of technology, the ethics of nondisclosure, and directions for practice and research.

Divergence From the Black DL Literature

The DL dynamic has traditionally been understood as a Black phenomenon. González observed that, although early press reports included young Latino men, they quickly faded from the discussion, and Latino HIV prevention scholarship did not substantively engage the issue [5]. The present analysis suggests that the Black DL literature cannot simply be extended to Latino populations but requires conceptual rethinking. Black men on the DL are generally understood as concealing a sexual identity they privately recognize [1,17], whereas the activo/pasivo system operates on different premises. A Latino man who assumes the insertive role and identifies as heterosexual is not suppressing a privately held gay identity but rather functioning within a cultural system in which sexual behavior, rather than the gender of one's partner, determines sexual identity [10,29]. Sexual identity in this context is relationally and situationally defined, such that a man may not identify as gay within family settings yet may do so within gay-identified social spaces. This context-dependent organization of identity is not adequately captured by a literature that conceptualizes nondisclosure as the concealment of a fixed private identity [3,5].

The structural conditions are also not interchangeable. Latino culture is collectivist and allocentric; the individual adjusts to the group rather than asserting separateness. Familismo ties disclosure to marriage, procreation, and family honor through obligations that carry different weight than the family pressures Black MSM navigate. Immigration, language, and the insularity of immigrant settlement communities, where gossip travels fast, add pressures that feed into one another. Aggregating these populations together under a minority MSM category has made it harder, not easier, to design interventions that actually fit either one.

The Impact of Technology

Technology has changed how DL culture operates and how the public talks about it. Social media, particularly TikTok, brought what was once a relatively insular topic into mainstream conversation. Users have created content explaining DL terminology, identifying signs of men living on the DL, and discussing the cultural dynamics of DL relationships within Hispanic and Black communities. Mainstream media have followed with recent coverage of the phenomenon [52]. Some of this content has opened up conversations about sexual identity and the pressures behind nondisclosure and, in that sense, has reduced stigma through visibility. However, much of it has been sensationalized or turned into entertainment, reinforcing stereotypes about bisexual men as deceptive threats to their female partners. For Latino men on the DL, increased visibility has expanded public awareness while making the cultural environment around them more hostile.

Geosocial networking apps have also reshaped DL sexual behavior. Grindr, Scruff, Sniffies, and similar platforms allow men to find sexual partners with considerable efficiency and without the risks of being seen in physical gay spaces such as bars or clubs. For men on the DL, the advantages are obvious: anonymous profiles, location-based matching, and control over what information gets shared. However, the research suggests that these advantages come with costs [53]. Latino MSM who meet partners online have been found to engage in higher rates of unprotected anal intercourse compared to those meeting partners through traditional venues, and researchers have noted that the efficiency of these platforms appears to be the primary driver of that risk [54]. Meta-analyses show that app users report more sexual partners and more high-risk sexual behavior than non-users [55].

The theoretical frameworks applied in this analysis help account for what these technologies do and do not accomplish. Apps allow men to act on desire without disrupting their concealed identity, but they do so by making compartmentalization more efficient. The concealment stress Pachankis [48] described is not reduced; it is managed at a distance. The transactional nature of most app-based encounters works against the development of intimate relationships or community ties, the kinds of connections that, over time, could provide a context for working through identity questions. There are also documented links between hookup apps and substance use, with studies finding that roughly one in 10 dating app users engage in drug use after being introduced to it through the platforms, and Sniffies users are significantly more likely to engage in chemsex compared to users on other platforms [56]. These findings are consistent with the syndemic dynamics described earlier, where substance use feeds into sexual risk, which increases HIV exposure, which generates stigma, which reinforces concealment. Men who perceive their sexual identity as stigmatized engage in strategic concealment and selective disclosure on these platforms, with location-based features representing both opportunities for connection and risks to privacy [57]. The technology provides men with a way to navigate the conditions described in this analysis without altering those conditions.

Ethics of Concealment

There are no clean answers to the ethical questions surrounding DL concealment. A man's right to privacy and self-protection exists alongside his female partner's need for information to make decisions about her own health and her own life. Research on nondisclosing behaviorally bisexual men shows that many of them see their same-sex behavior as nobody else's business, too personal to share [58,59]. From that standpoint, keeping it private is not lying. It is choosing not to volunteer information about something deeply personal.

That reasoning becomes harder to sustain inside an intimate relationship where a woman believes she is in a monogamous heterosexual partnership. She cannot make informed decisions about her sexual health or her future without knowing what is happening. The consequences are particularly serious with respect to HIV transmission. Studies have found that many HIV-positive individuals do not disclose their serostatus to sexual partners and that those who do not disclose are no more likely to use condoms consistently than those who do [60]. The American Medical Association has acknowledged this challenge, supporting protections for physicians' clinical judgment on partner notification while encouraging efforts to persuade HIV-positive patients to inform those at risk [61].

However, framing nondisclosure solely as a moral failure on the part of individual men overlooks the structural and cultural conditions documented throughout this review. Nondisclosure among Latino men on the DL functions primarily as a strategy for managing stigma, a means of avoiding anticipated rejection, violence, loss of family support, and social exile. The ethical dimensions of this behavior must be considered alongside the reality that, for many of these men, disclosure carries the risk of physical harm, financial loss through family rejection, and severe psychological consequences from community condemnation. Heteronormative cultural expectations, religious condemnation, and widespread homophobia within both Latino communities and mainstream society have produced the conditions under which concealment becomes a means of survival. As Boykin argued, the racism, poverty, and homophobia that constrain men's choices are more appropriate targets for intervention than the men themselves [62]. This position is consistent with the broader argument of this review, that the cultural and structural conditions producing concealment, rather than the individuals who practice it, should be the primary objects of research and intervention.

Limitations

This review has several limitations. The search was limited to sources in English and Spanish, which may have excluded relevant work published in Portuguese or other languages spoken across Latin America. Although multiple databases were searched and SANRA guidelines were followed, some relevant studies may not have been captured, particularly unpublished dissertations or grey literature not indexed in the databases used. The focus was restricted to US-based Latino populations, and the findings may not apply to Latino MSM in other national contexts. Within the US Latino population, differences by country of origin, immigration generation, language dominance, or geographic region could not be examined because the literature has not produced enough disaggregated data to support that level of analysis. Finally, nearly all of the studies available for review were cross-sectional, which constrained the ability to draw conclusions about how DL behavior develops or changes over time.

Clinical Implications and Future Directions

The findings of this review have implications for how clinicians approach sexual health with this population. Because research on queer populations suggests that disclosure of one's queer identity is correlated with better mental health, many clinicians assume a pro-coming-out stance and will overtly or subtly encourage non-heterosexuals to disclose their orientations. The problem with this assumption, as the research cited throughout this work demonstrates, is that many of these men do not identify as gay or bisexual and may not conceptualize their same-sex behavior as constituting a sexual identity at all, making the very framework of "coming out" incongruent with their self-understanding. Beyond this, Latino men on the DL face identity management challenges that make open disclosure genuinely dangerous in ways that differ from those experienced by White MSM. Clinicians are advised to be aware of these issues when practicing identity-affirmative counseling, since there is a great deal at stake when these men begin disclosing.

Clinical environments need to be structured so that disclosure feels safe rather than threatening. This goes beyond asking about sexual behavior during intake. Providers need training in how machismo, familismo, and religious influences shape Latino men's decisions about disclosure, and they need to recognize that silence about same-sex activity is not deception but a response to legitimate fears of family rejection, community ostracism, and loss of standing. Routine universal HIV testing, offered as standard practice rather than in response to disclosed risk behavior, removes the need for men to identify as having same-sex partners to access prevention. Health practitioners involved in HIV prevention and behavioral health interventions should seek to work with relationship-oriented cultural factors, such as personalismo, machismo, and familismo, as experienced by Latino sexual minority men, rather than treating these values as obstacles to be overcome. Tailoring interventions to acknowledge these men's experiences of these cultural values could improve engagement and effectiveness, particularly as these values shape interactions within both ethnic and sexual identity communities.

Intake forms and clinical language built around gay, bisexual, and straight categories will miss men whose sexual lives are organized differently. Clinicians working with this population need to ask about behavior without requiring men to adopt identity labels that carry cultural meanings they reject. Interventions designed for openly gay-identified men and then superficially adapted are unlikely to reach nondisclosing Latino MSM. Addressing HIV risk in isolation from immigration barriers, language access, poverty, and cultural stigma will produce limited results because these conditions reinforce one another. Interventions need to move beyond individual counseling to incorporate family education where appropriate, community-level stigma reduction, and structural changes to healthcare access. Particular attention is needed in emerging Latino communities in the Midwest and rural South, where culturally congruent resources for Latino MSM are largely absent.

Future research should investigate the development, implementation, and evaluation of sexual health interventions tailored for nondisclosing Latino MSM to determine their effectiveness in meeting the needs of this population. Such interventions should be co-designed with community members and should address not only individual risk behavior but also the family systems, religious institutions, and community norms that constrain men's choices. Longitudinal studies following men over extended periods are needed to understand how DL behavior evolves across the life course and what factors precipitate disclosure or continued concealment. Researchers may want to consider conducting qualitative phenomenological studies that examine the lived experiences of these men as they relate to Latino cultural factors, concealment, and health outcomes. Research exploring heterogeneity within the Latino population is also needed, examining how DL behavior differs by national origin, generational status, language preference, and geographic region. Finally, implementation research is needed to address the practical challenges of reaching this population and keeping them engaged in care.

## Conclusions

This review applied three complementary theoretical frameworks to examine DL behavior among Latino men in the United States and found that the phenomenon cannot be adequately explained through any single model alone. The interaction of machismo, familismo, religion, and immigration status produces conditions under which nondisclosure functions as a strategy for preserving family belonging and masculine standing rather than as a failure to disclose a concealed identity. The concealment-specific model, SCT, and syndemic theory each address a different dimension of this experience, and their convergence on the same population suggests that future interventions must engage the cultural, psychological, and structural levels simultaneously if they are to produce meaningful change. The findings from this review lay the groundwork for empirical research that examines the lived experiences of nondisclosing Latino MSM on their own terms, within their own cultural logic, and with attention to the conditions that sustain concealment rather than to the men who practice it.
